# Minimal mechanistic component of HbYX-dependent proteasome activation that reverses impairment by neurodegenerative-associated oligomers

**DOI:** 10.1038/s42003-023-05082-9

**Published:** 2023-07-14

**Authors:** Janelle J. Y. Chuah, Tiffany A. Thibaudeau, David M. Smith

**Affiliations:** 1 Department of Biochemistry and Molecular Medicine, West Virginia University School of Medicine, 64 Medical Center Dr., Morgantown, WV USA; 2 Department of Neuroscience, Rockefeller Neuroscience Institute, West Virginia University, Morgantown, WV USA

**Keywords:** Proteasome, Alzheimer's disease

## Abstract

The implication of reduced proteasomal function in neurodegenerative diseases combined with studies showing the protective effects of increasing proteasome activity in animal models highlight the need to understand the capacity for proteasome activation by small molecules. The C-terminal HbYX motif is present on many proteasome binding proteins and functions to tether activators to the 20S core particle. Previous studies have shown that peptides with a HbYX motif can autonomously activate 20S gate-opening to allow protein degradation. In this study, through an iterative process of peptide synthesis, we design a HbYX-like dipeptide mimetic that represents only the fundamental components of the HbYX motif. The mimetic robustly induces gate-opening in archaeal, yeast, and mammalian proteasomes. We identify multiple proteasome α subunit residues in the archaeal proteasome involved in HbYX-dependent activation. When stimulated by the mimetic, the mammalian 20S can degrade unfolded proteins such as tau. Findings using our peptide mimetic suggest the HbYX-dependent mechanism requires cooperative binding in at least two intersubunit pockets of the α ring. Most significantly, our peptide mimetic reverses proteasome impairment by neurodegenerative disease-associated oligomers. Collectively, these results validate HbYX-like molecules as having robust potential to stimulate proteasome function, which are potentially useful for treating neurodegenerative diseases.

## Abstract (web summary)

An HbYX-like dipeptide induces gate-opening in archaeal, yeast, and mammalian 20S proteasomes, stimulates proteasome activity, and reverses inhibition of the proteasome by toxic protein oligomers that are implicated in various neurodegenerative diseases.

## Introduction

**Fig. 1:**
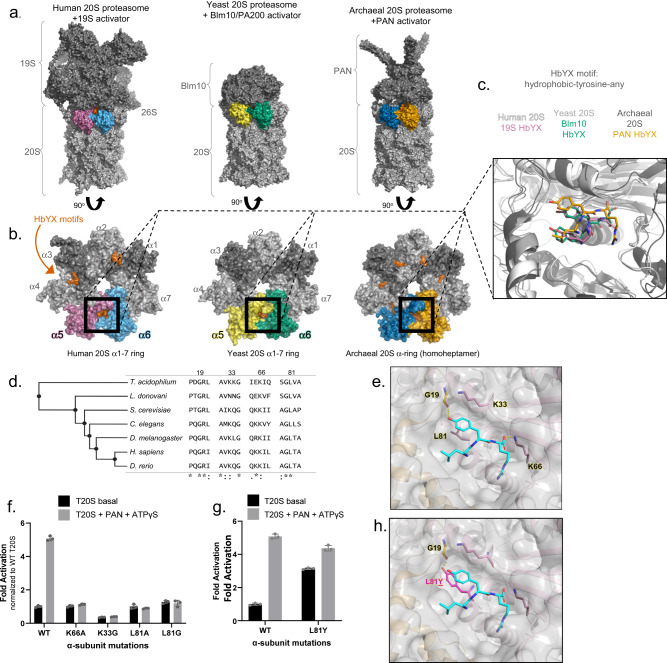
Identifying residues in the α intersubunit pocket critical for HbYX-dependent gate-opening. **a** Surface representation of 20S proteasomes in complex with activators [Human 26S (PDB 6msk), yeast 20S+Blm10 (PDB 4v7o), archaeal 20S + PAN (PDB 6hed)]. HbYX motifs visible are colored red-orange and adjacent α-subunits of the visible HbYX motif are shown in various colors. **b** Surface representation of the 20S α rings (from a) down the center axis with activator caps removed. Proteasome activator C-termini HbYX residues are shown in red-orange (surface). **c** Overlay of H20S and Y20S intersubunit pockets α5/6 and T20S intersubunit pockets α/α (cartoon) from B with HbYX motif residues (sticks). Crystal structure of PAN C-terminus (PDB 3ipm) is shown in place of Cryo-EM PDB 6hed. Images were rendered with PyMOL. **d** Multiple sequences alignment of the T20S α subunit with various eukaryotic α6 subunits generated with CLUSTAL OMEGA (1.2.4). **e** Conserved residues interacting with bound HbYX motif (sticks) in the T20S intersubunit pocket (PDB 3ipm). PAN HbYX motif (LYR) shown in cyan (stick). **f** Rate of substrate degradation (fluorogenic nonapeptide LFP) by the wild type (WT) T20S proteasome (0.14 nM) or K66/K33/L81 mutants incubated with or without PAN (with ATPγS). Stimulation of gate opening was measured by the increase of LFP hydrolysis (rfu/min) relative to WT 20S without PAN. **g** Experiments with T20S proteasome (0.35 nM of wild-type or L81Y mutant) performed same as in (**f**). **h** Same as (*E*), with L81 mutated to tyrosine (magenta stick). Images in e and h were rendered with PyMOL. Data (means) are representative of three or more independent experiments each performed in triplicate. Error bars represent ± standard deviation.

The proteasome is a key component of the ubiquitin-proteasome system (UPS), responsible for removing damaged or unneeded proteins and regulating major cellular processes^1^. Regulation by proteasome activators (PAs) are critical to ensure that only proper proteins are degraded. Dysregulation of the proteasome has been implicated in several neurodegenerative diseases (NDs), often characterized by impairment of proteasome function^2–7^. The purose of this study is to define the minimal proteasome activating elements of HbYX-dependent proteasome activators and to provide a molecular framework to guide drug-discovery approaches aimed at activating proteasomal degradation to treat ND which, consequently, is expected to help elucidate how ND-associated oligomers impair proteasome function. The core particle of the eukaryotic proteasome, also referred to as 20S (Fig. 1a), consists of four stacked heteroheptameric rings (α-β-β-α) with a central pore for substrate entry. The β rings consist of seven subunits (β1-7), three of which harbor protease sites. The two α rings also consist of seven subunits (α1-7). Substrate entry in the 20S is regulated by the gate, which primarily consists of the N-terminus of α 2, 3, and 4 extending over the central pore thus closing off this barrel-shaped structure^8^. The closed gate conformation blocks the central pore and prevents proteins from entering the 20S to be degraded. The N-terminus of each α subunit carries a YDR (tyrosine-aspartate-arginine) motif that interacts with neighboring N-termini to stabilize the closed state of the gate^8^. These N-termini extensions can also change their conformation to an “open” state, whereby they point up and outwards from the α ring pore, which is stabilized by an alternate interaction from the YDR motif ^9^. Additionally, truncation of α3’s N-terminus (α3∆N), which act as a central lynchpin to stabilize the closed state, generates a constitutively open (active) 20S that is highly capable of degrading unstructured proteins^8,10^. Assessed by NMR, the basal kinetics of archaeal 20S gate-opening has been suggested to undergo open/close fluctuations on a time scale of seconds^11,12^. Although the kinetics have not yet been measured for the mammalian 20S (M20S), the presence of basal protein or peptide hydrolysis activities suggest that the M20S gate also fluctuates between these states, though likely slower.

While the 20S gate typically favors the closed state, binding of proteasome regulatory complexes (Fig. 1a) to the α ring can trigger conformational changes that cause gate-opening, allowing the 20S to accept unfolded and linearized substrates^13,14^. Two different mechanisms of 20S gate-opening have been described, the HbYX-dependent and the 11S family-dependent mechanisms. Thus far, most studies on the HbYX-dependent mechanism focus on the 19S, also known as PA700 or the Regulatory Particle (RP), which associates with the 20S to form the 26S complex that degrades ubiquitinated proteins (Fig. 1a). The 19S consists of a base subcomplex, which is primarily composed of a heterohexameric ring of ATPases (Rpt1-6), and a lid subcomplex, which contains ubiquitin binding and processing subunits. The 19S has been shown to stimulate gate-opening by the docking of the Rpt1-6 C-terminal tails, some of which contain the HbYX motif, in the intersubunit pockets of the 20S α ring^15^. The use of C-terminal tails to associate with the 20S has also been observed in other Proteasome Activators (PAs) including PAN (proteasome-activating nucleotidase; the archaeal homolog of the 19S), PA200/Blm10 (Fig. 1a–c), and 11S activators.

Not all C-terminal tails of PAs induce gate-opening when bound to the 20S, as evident by the observation that peptides corresponding to the C-terminus of PA26 (a member of the 11S family) cannot induce gate-opening autonomously^16^. Conversely, peptides corresponding to the C-terminus of Rpt2, Rpt3, Rpt5, PAN, and PA200/Blm10 can autonomously induce gate-opening^16,17^. All the peptides that induce gate-opening carry the HbYX motif, which has been shown to be essential for allowing these complexes to associate with the 20S^15,16^. The C-terminal HbYX motif binds into pockets formed by the interface of the α subunits in the 20S, called intersubunit pockets (Fig. 1b, c). Whereas the C-termini of PAN homohexameric ATPases all have a HbYX motif, only the C-terminus of Rpt2, 3, & 5 from the 19S heteromeric ATPases have the HbYX motif and Rpt1 has a partial HbYX motif, lacking the Hb residue. The roles that the C-termini of Rpt4 and Rpt6 (which lack the HbYX motif) play in the association of the 19S-20S and 20S gating regulation are unclear but have been observed bound to intersubunit pockets via crosslinking and cryo-EM^18–20^. The binding of HbYX-peptides to intersubunit pockets, structurally distant from the gating residues, results in gate conformational change. This demonstrates that the HbYX motif functions allosterically, and likely induce substantial conformational changes in the α subunits that in turn, affect the conformation of gating residues^16,21^.

In contrast, the family of 11S activators does not carry a HbYX motif on their C-terminal tails. Their mechanism of gate-opening is relatively well-known compared to the HbYX-dependent mechanism. They associate with the 20S, using their C-termini to dock in the α intersubunit pockets, similar to the HbYX-dependent activators. However, to trigger gate-opening, the 11S family relies on “activation loops” that interface directly with the base of the gating N-termini in the pore of the α ring^22,23^. These activation loops appear to sterically repel a reverse turn proline (Pro17) at the base of the gating residues shifting it by <1 Å, which is sufficient to disrupt the closed state and stabilize the open state^9^. Interestingly, minimal conformational changes in the α subunits (excluding gating regions) are necessary for gate-opening by the 11S activators, as shown by the crystal structure of PA26-20S proteasome^9^. It is evident that the two families of PAs (HbYX-dependent and HbYX-independent) use different strategies to induce 20S gate-opening. However, the regulatory ATPase-20S complex and its HbYX motif for binding and gate opening are conserved in all organisms with proteasomes including archaea and eukaryotes. Based on this structural and functional conservation we expect that the HbYX-dependent mechanism of gate-opening would also be conserved in archaeal and eukaryotic 20S proteasomes. Although the location and effect of HbYX-binding has been investigated, the molecular mechanism of HbYX-dependent gate opening appears to be surprisingly complex and remains unsolved.

Recently, we showed that ND-associated proteins (i.e., amyloid-β, α-synuclein, and huntingtin) can fold into a common conformation that inhibits 20S and 26S proteasomes^21^. We also learned that these soluble oligomers (A11+) inhibit the 20S by allosterically stabilizing the closed gate conformation^21^. This negative allosteric regulation by such toxic^24^ oligomers appear to be mechanistically coupled to the HbYX-dependent mechanism of 20S gate-opening^21^, suggesting that these oligomers and the HbYX motif are allosteric regulators of the same gating mechanism. Therefore, we hypothesize stimulating 20S activity via the HbYX-dependent mechanism will antagonize impairment by ND related oligomers, which could restore proteasome function and stimulate protein degradation, thereby potentially providing a therapeutic approach for ND.

In this study, we designed a small molecule that functionally emulates the HbYX (hydrophobic-tyrosine-variable C-terminal residue) motif-dependent mechanism of proteasomal gate-opening and robustly activates the archaeal, yeast, and mammalian proteasomes. Using our small molecule as a research tool, we elucidate the fundamental requirements necessary for the HbYX motif to induce gate-opening. Moreover, this proteasome-activating small molecule can reverse inhibition of the proteasome by toxic soluble protein oligomers implicated in neurodegenerative disease, such as amyloid-β, α-synuclein, and huntingtin exon1.

## Results

### Identifying essential structures for HbYX dependent gate-opening

The HbYX motif is highly conserved and found in proteasome activators across archaea, eukaryotes and even some actinobacteria (Fig. 1a–c). Through mutagenesis and structural analyses, we found that the intersubunit pockets contain multiple conserved residues, some of which are already known to be important for gate activation (i.e., Pro17, Lys66 from *Thermoplasma acidophilum*)^9,16,25^ (Fig. 1d). To better understand how the HbYX motif interacts with the 20S, we aligned HbYX-bound intersubunit α pockets from human (PDB 6msk, cryo-EM), yeast (PDB 4v7o, crystallography), and archaeal proteasomes (PDB 6hed, cryo-EM) (Fig. 1b, c). We observed in these structures that the HbYX motif from these three diverse species binds to the 20S intersubunit pockets in highly similar orientations, with three distinct and shared interactions (Fig. 1c–e): (1) The C-terminal carboxylic acid of the motif is directed towards the conserved lysine (K66 in archaea), which is already known to be required for 20S proteasome-activator complex formation, (2) the penultimate tyrosine’s hydroxyl group hydrogen-bonds with the backbone carbonyl of G19 and is oriented toward the proline reverse turn (located at the base of the gate), and (3) the hydrophobic (Hb) group contacts a hydrophobic pocket in the neighboring α subunit.

We investigated the effect of mutating three of the conserved residues that are positioned to interact with the HbYX-motif (Fig. 1f) in the *T. acidophilum* 20S (T20S). We chose the T20S because it is a symmetric homoheptamer, which facilitates simultaneous mutagenesis in all 7 intersubunit pockets. We then tested the activation of the T20S mutants by PAN (measuring LFP nonapeptide degradation) since PAN uses the HbYX-dependent mechanism (Fig. 1f). Consistent with previously published results^16^, K66A mutation prevented PAN activation of the T20S. The basal activity of T20S-K66A was similar to the wild type (WT). Structural studies showed that the aliphatic portion of the K33 side chain and L81 are positioned on either side of the HbYX tyrosine ring, interacting hydrophobically to hold the HbYX tyrosine ring in place (Fig. 1e). When K33 was mutated to glycine, the mutant T20S could no longer be stimulated by PAN (Fig. 1f). Also, T20S-K33G basal activity was about half of the wild type. Similarly, the mutation, L81A (located directly under the HbYX tyrosine) prevented PAN-mediated proteasome activation. While our primary interpretation of the results described thus far is that our mutations (K66A, K33G, etc.) prevents activation by PAN and/or gate-opening, an alternative interpretation is that the mutations could simply prevent PAN from binding to the T20S. However, the lack of stable binding between T20S and PAN limits our ability to experimentally distinguish between the two interpretations. Therefore, based on prior structures and these biochemical results, we conclude that K33 and L81 are both important for stabilizing and, likely, orienting the HbYX tyrosine in the intersubunit pocket.

Considering the importance of HbYX tyrosine hydrogen bonding with the backbone carbonyl of G19, we investigated whether substituting tyrosine for L81 (L81Y) could mimic the effect of a bound HbYX motif, as the mutation would place a tyrosine ring in similar space as the HbYX tyrosine (Fig. 1g, h). We found that proteasomes with the L81Y mutation (T20S-αL81Y) had elevated basal activity, relative to WT (Fig. 1g), suggesting that the mere placement of a tyrosine into this location is sufficient to partially induce gate opening, similar to HbYX binding. In agreement with this conclusion, the addition of PAN at saturating concentration to T20S-αL81Y proteasomes resulted in a further 30% increase in activation to near WT-like activation (Fig. 1g), indicating the L81Y gate was not completely opened by mutation alone. These results demonstrate that the proper placement of a tyrosine alone in all intersubunit pockets of the archaeal 20S proteasome is sufficient to induce conformational changes leading to at least partial 20S gate opening.

### Generating a minimal HbYX motif-based peptide to stimulate gate opening

**Fig. 2:**
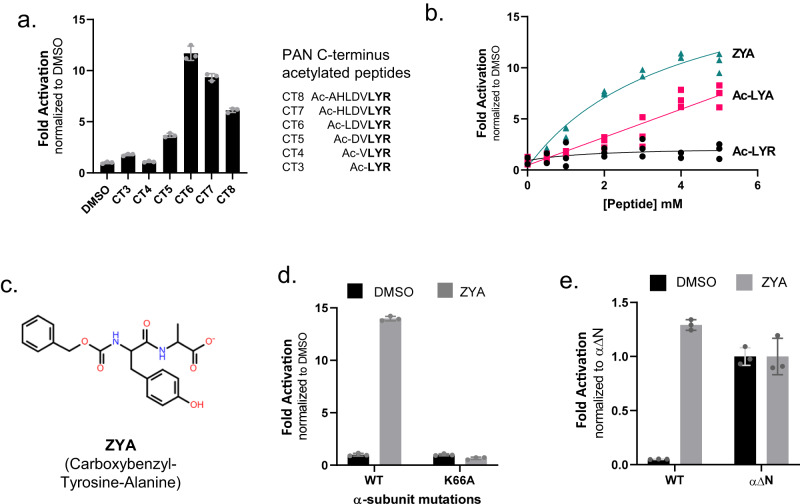
Generation of a minimal HbYX motif mimetic capable of opening the archaeal 20S proteasome’s substrate gate. **a** N-terminally acetylated peptides (200 µM) were incubated with 7 nM WT T20S proteasomes and LFP. Stimulation of gate opening was measured by the increase of LFP hydrolysis (rfu/min) relative to WT 20S with DMSO. Sequences of peptides are shown. **b** Dose response of peptides (Ac-LYR, AC-LYA, ZYA) with 7 nM WT T20S proteasomes and LFP. LFP degradation rate (rfu/min) is normalized to DMSO. **c** Structure of ZYA peptide mimetic. **d** ZYA (2.5 mM) incubated with 7 nM WT or K66A T20S and LFP. LFP degradation rate (rfu/min) normalized to DMSO. **e** ZYA (2.5 mM) incubated with 7 nM WT or gateless (α∆N) T2S0 and LFP. LFP degradation rate (rfu/min) normalized to αΔN. Data (means) are representative of three or more independent experiments each performed in triplicate. Error bars represent ± standard deviation.

Since the L81Y mutation was able to induce partial gate opening, we hypothesized that small peptides mimicking the HbYX-motif could activate 20S gate opening. Our previous study in 2007 showed that only peptides >7 residues long with a C-terminal HbYX motif (corresponding to the PAN’s C-terminus) could induce gate opening^16^. However, this was determined with peptides that had unmodified N-termini. We suspected that the charged N-terminus may prevent shorter HbYX peptides from binding to the intersubunit α pockets. To test this hypothesis, we synthesized the same peptides corresponding to the PAN C-terminus from three to eight residues in length (CT3-8), except with an acetylated N-terminus. N-acetylated PAN C-terminus peptides shorter than seven residues activated the 20S (Fig. 2a). Notably, we observed that N-acetylated PAN C-terminus peptide six residues in length (CT6) activated the 20S over 10-fold greater than the control; even the 3-residue peptide (CT3) had (~2-fold) gate-opening activity (Fig. 2a).

Based on the successful gate-activation of PAN CT3 (Ac-LYR) after a single modification (acetylated N-terminus), we investigated whether additional modifications would further improve the efficacy of the peptide. Our previous study showed that the substitution of alanine for arginine does not affect PAN-CT8 peptide efficacy to bind α intersubunit pockets and open the gate^16^. We therefore substituted an alanine in place of the bulky arginine residue, which resulted in greater activating efficacy, compared to Ac-LYR (Fig. 2b). Attempting to further minimize the size of the molecule, we chose carboxybenzyl (Z) as an N-terminal blocking group that could eliminate the N-terminal charge and mimic the Hb group (of the HbYX motif). Combining these modifications resulted in a dipeptide with a hydrophobic group preceding the N-terminus of tyrosine (Z-Tyr-Ala or ZYA) (Fig. 2c). We compared ZYA against Ac-LYR and Ac-LYA and observed significant improvements in T20S activation (Fig. 2b). ZYA could substantially activate T20S activity, but its affinity was poor (see below). While ZYA could activate T20S ~13 fold, it could not stimulate gate-opening in T20S-K66A to any extent (Fig. 2d), demonstrating that ZYA requires K66 in the intersubunit pocket as expected. This suggests that activation of T20S by ZYA occurs through interactions similar to those responsible for activation by PAN. To rule out the possibility that ZYA might be activating the protease sites rather than inducing gate opening, we also tested the capacity of ZYA to stimulate the αΔN-T20S, which lacks gate residues and is constitutively open. ZYA did not stimulate the activity of αΔN-T20S to any extent (Fig. 2e). Together, these results are consistent with ZYA activating the 20S by inducing gate-opening, similar to the HbYX motif on which it was based.

### ZYA activates mammalian proteasomes

**Fig. 3:**
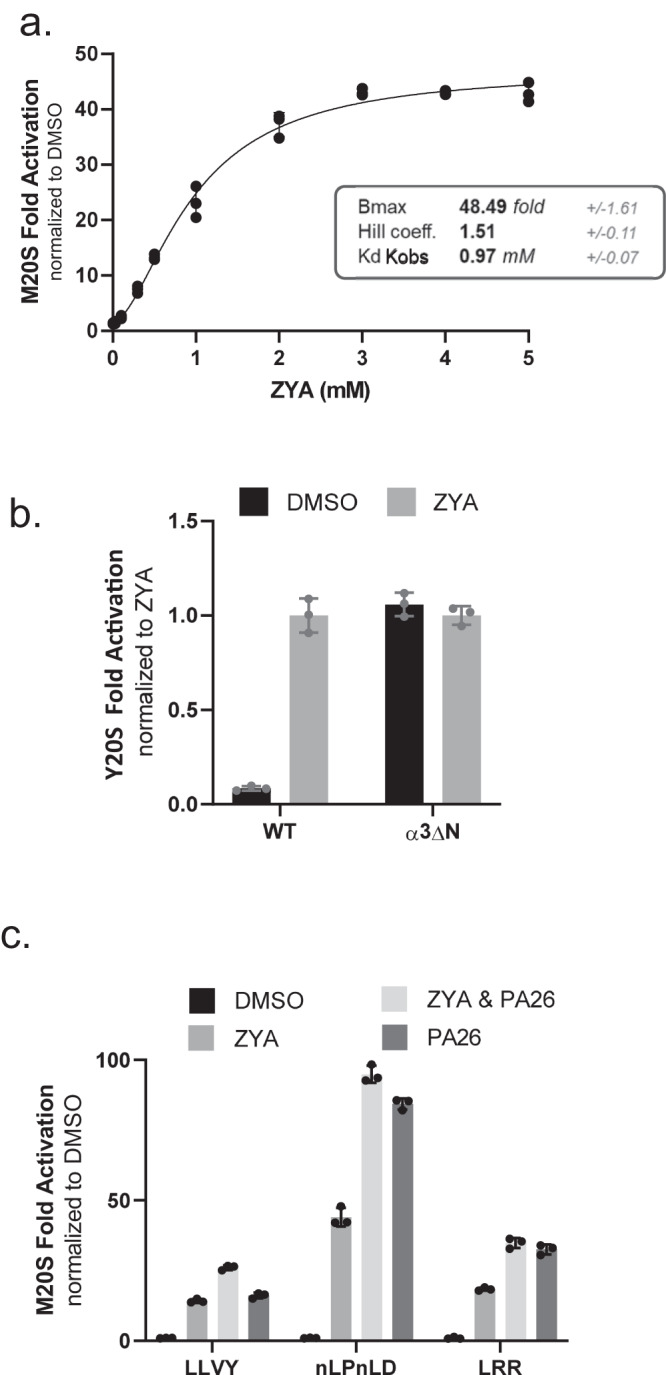
ZYA robustly stimulates gate-opening in yeast and mammalian proteasomes. **a** Dose response of ZYA with mammalian 20S proteasomes (0.5 nM) and nLPnLD-AMC as substrate. Proteasome activity is normalized to DMSO control. Means were fit to the Hill equation, and equilibrium binding coefficients are shown. **b** WT and gateless (α3∆N) yeast 20S proteasomes (0.5 nM) incubated with or without ZYA (2.5 mM) and nLPnLD-amc. **c** Mammalian 20S proteasomes (0.5 nM) alone or with ZYA (2.5 mM), or PA26 (55 nM), or both (see graph key). Proteasome activity measured using three different fluorogenic substrates preferentially cleaved by different 20S protease sites (LLVY-amc, chymotrypsin-like, nLPnLD-amc, caspase-like; LRR-amc, trypsin-like). Data (means) are representative of three or more independent experiments each performed in triplicate. Error bars represent ± standard deviation.

We sought to determine if our findings with ZYA on the *Thermoplasma* 20S would translate to the mammalian system; therefore, we tested Ac-LYR, Ac-LYA, and ZYA on M20S (Supplemental Fig. 1a). In agreement with the T20S outcomes, we observed significant improvements in gate-opening efficacy with each modification. ZYA exhibited the greatest effect on the M20S, and a concentration response with ZYA showed nearly 50-fold activation at saturating levels (Fig. 3a vs Supplemental Fig. 1a). Although ZYA can stimulate the M20S activity robustly, it has low affinity with a k_obs_ of ~1 mM (Fig. 3a), which is highly similar to its affinity for the T20S (Fig. 2b). To confirm that ZYA activates via gate opening in eukaryotic proteasomes, we asked if ZYA could activate the yeast open-channel mutant (α3∆N) 20S. We found that ZYA could not activate it at all but did activate WT yeast 20S (Y20S) as expected (Fig. 3b), consistent with analogous experiments in the archaeal system. Interestingly, the saturation curve for ZYA-induced proteasome activity for the M20S gave a cooperative binding curve with a significant hill coefficient of 1.5 ± 0.1 (Fig. 3a). This indicates that ZYA binding is cooperative and that binding to more than one site occurs during the allosteric induction of gate opening. This is consistent with published cryo-EM structures of the 26S^18,19^ that showed multiple C-termini binding before the open-gate state is fully stabilized. While the minimal number of HbYX motifs required to induce gate opening is unknown, the hill coefficient of 1.5 suggests that a minimum binding of two molecules is involved. Alternatively, the first binding of the HbYX motif to any intersubunit pocket of the T20S influences the binding of the second binding of the motif on the α ring of the opposite side of the T20S. However, neighboring subunit allostery is more plausible since the binding of the 26S ATPases all occur on adjacent pockets in a single α ring.

We next tested the ability of ZYA to stimulate the M20S to hydrolyze three different peptide substrates that are preferentially cleaved by 20S’s three different protease sites (suc-LLVY-amc, β5; Ac-nLPnLD-amc, β1; Boc-LRR-amc, β2). We observed a significant 10- to 50-fold increase, in the hydrolysis rate of all three peptide substrates (Fig. 3c, compared to DMSO), which is expected for a gate opener. In addition, to determine how well ZYA was able to induce gate-opening, we compared it to M20S activation by PA26. Saturating PA26 concentrations typically stimulate M20S activity by 30-100-fold, depending on the basal activity of the 20S preparation. We found that ZYA was able to stimulate the M20S similar to that of PA26: ~50-fold activation by ZYA and ~90-fold for PA26 (for Ac-nLPnLD-AMC). In addition, when we combined ZYA and PA26 in a single reaction there was no synergy between these activators. Instead, there was a slight decrease in activity, relative to PA26 alone, which is likely due to expected competition between ZYA and PA26 binding to the intersubunit pockets. Collectively, these results demonstrate that ZYA is a highly effective and robust gate-opening activator of the M20S proteasome from archaea, yeast and mammals. These results support the hypothesis that this HbYX peptide mimetic functions analogously to the highly conserved HbYX motif.

### ZYA stimulates protein degradation by the mammalian 20S proteasome

**Fig. 4:**
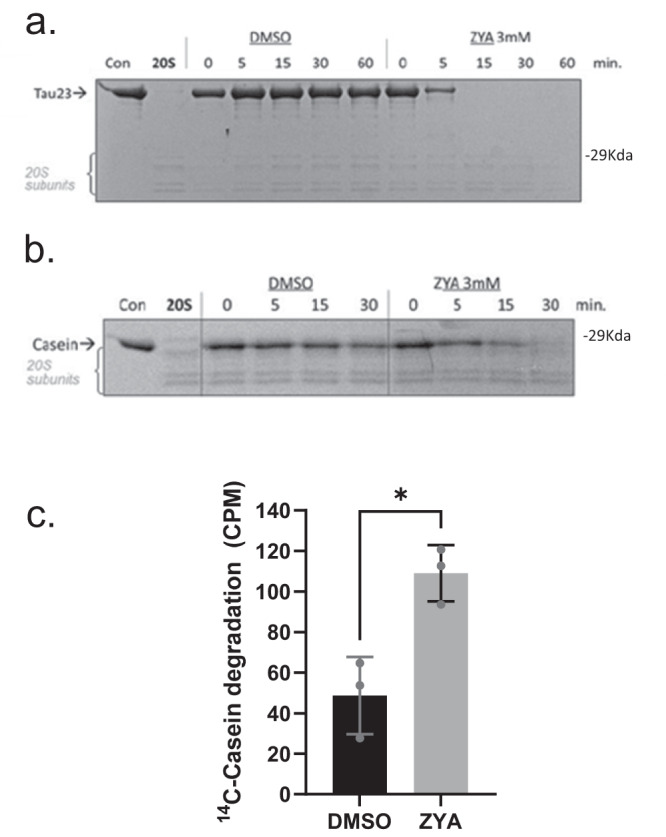
ZYA stimulates mammalian 20S to degrade unstructured protein substrates. **a** Mammalian 20S proteasomes (100 nM) incubated with tau23 (2uM). At the indicated times, the reaction was quenched by the addition of SDS loading buffer and separated by SDS-PAGE. Proteins visualized with Coomassie brilliant blue. Gels are representative of three independent experiments. 20S proteasome subunits are indicated with brackets to serve as loading controls for each sample. **b** Same as a, except with β-casein (1 uM). **c** ^14^C casein was incubated with mammalian 20S proteasome similar to D for 30 min at 37^o^C.  Acid soluble counts after TCA precipitation were quantified via scintillation to show the generation of peptide products. *p*-value: 0.011. Data (means) are representative of three or more independent experiments each performed in triplicate. Error bars represent ± standard deviation.

We have demonstrated that ZYA robustly activates peptide hydrolysis via gate-opening but what about that 20S’s capacity to degrade unstructured proteins? To answer this question, we asked if ZYA could stimulate the M20S proteasome to degrade tau23 (a truncated tau protein that is found in brain) and the model unstructured protein—casein. ZYA significantly increased the degradation of both proteins, as visualized by SDS-PAGE with Coomassie stain (Fig. 4a, b). Tau and casein appeared to be completely degraded within the first 15 min and 30 min, respectively. In support of these results, we also measured the peptides generated from the degradation of ^14^C-casein by the 20S in solution (Fig. 4c) as measured by acid-soluble counts. In agreement with the gel-based protein degradation assay, ZYA significantly increased the number of soluble peptides from the degradation of ^14^C-casein. These results clearly demonstrate that ZYA can robustly stimulate the degradation of unfolded proteins.

### Probing structure activity relationships of the ZYA gate-opening compound

**Fig. 5:**
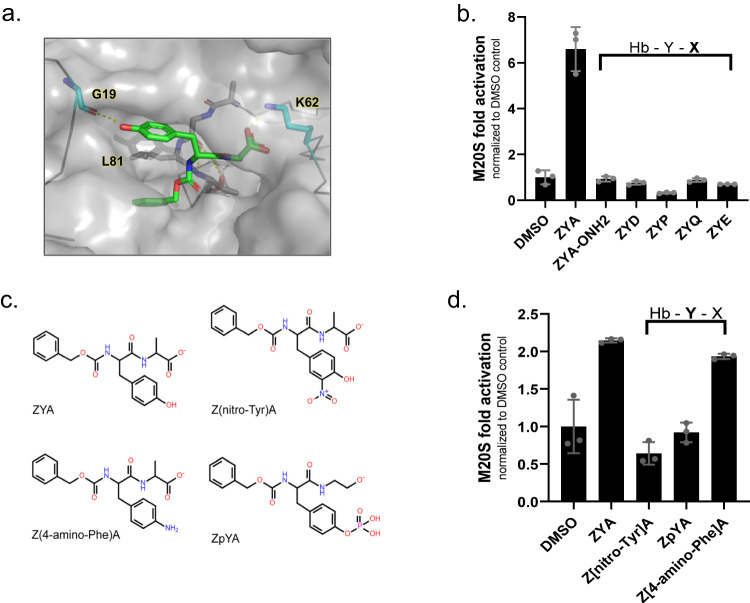
Probing structure activity relationships of the ZYA gate-opening compound. **a** ZYA (green sticks) docked in intersubunit pocket between α5&6 in human 20S. Image rendered in PyMOL. **b** Mammalian 20S proteasome (0.5 nM) activity (nLPnLD-amc hydrolysis, rfu/min) with the indicated ZYA derivatives (500 μM) with variations in the “X” position that were found to be deleterious to ZYA activity. Proteasome activity is normalized to DMSO. **c** Structures of derivatives tested in (**d**). **d** Mammalian 20S proteasome activity (nLPnLD-amc hydrolysis, rfu/min) with the indicated ZYA derivatives at a low binding concentration of 100 μM. Proteasome activity is normalized to DMSO. Data (means) are representative of three or more independent experiments each performed in triplicate. Error bars represent ± standard deviation.

To elucidate how ZYA might be binding to the human proteasome, we computationally docked ZYA in the human α5/6 intersubunit pocket, where the HbYX motif of Rpt5 binds. Our docking results (Fig. 5a) suggest that ZYA binds in a similar configuration to the HbYX motif of various PAs (Fig. 1c). To evaluate the specificity and HbYX motif-like requirements of ZYA for the M20S, we proceeded to probe ZYA’s structure/function relationships and efficacy through chemical modifications (Fig. 5b–d). First, we asked if additional negative charges in the “X” position of the HbYX motif could be tolerated by replacing alanine with acidic residues. We found that ZYE and ZYD failed to activate the M20S (Fig. 5b). We also tested if causing a backbone torsion constraint (ZYP) and a polar group (ZYQ) might stabilize the peptide’s structure and binding, but they too abrogated M20S stimulation activity (Fig. 5b). Prior studies and sequence conservation showed that small, medium, and large aliphatic, and basic residues could all be tolerated in the “X” position, thus the “X—variable” designation^16^. Our data with the ZYA also suggests there are some limitations (i.e., negative charges) in this position. Prior studies^9,16,25^ also showed the HbYX motif’s terminal carboxy group forms an ionic bond with K66. To validate that this was important for ZYA function in M20S, we blocked its C-termini carboxyl with a NOH2 group (ZYA-[NOH2]). Carboxy blocking completely abrogated ZYA activity (Fig. 5b), as expected for canonical HbYX motif function. We next considered that bridging of the intersubunit pocket by ZYA (i.e. binding between G19 or one subunit and K66 of the neighboring subunit, Fig. 5a) as suggested by the binding of the motif in published structures, and our docked model (Fig. 5a), could contribute to inducing conformational changes that cause gate opening. To test this hypothesis in the M20S, we modified the tyrosine of ZYA to lengthen the “bridging” distance by adding a nitro or phospho group to the tyrosine hydroxyl, which would lengthen the bridging distance by 1 or 2 bonds respectively (Fig. 5c). Both ZpYA and Z(nitro-Tyr)A failed to activate the 20S (Fig. 5d), supporting the bridging length requirement between the tyrosine hydroxyl and carboxy C-termini of ZYA. This conclusion is further supported by the fact that Z(4-amino-Phe)A still activates the 20S to a similar extent as ZYA (Fig. 5d), since it is similar in bridging length to ZYA.

### Comparing ZYA to known proteasome-activating small molecules

Recently, several studies have been published on the potential of small molecules to activate the 20S proteasome though their mechanisms of activation are still not clear. We compared how ZYA, designed to mimic the HbYX motif, performs relative to some of these small molecules (AM-404, TCH-165, Fluspirilene (FLP)^26,27^). The other small molecules exhibited higher binding affinity relative to ZYA (with K_obs_ values in the micromolar range; Table 1, Supplemental Fig. 1b). However, as indicated by V_max_ values, none of them activated the M20S to the extent that ZYA was capable of even at saturating concentrations (Table 1; Supplemental Fig. 1b). Interestingly, none of the other compounds significantly activated the T20S or any of its variants (e.g., WT, αΔN, K66A) (Supplemental Fig. 2). Notably, ZYA activates WT T20S, but not the T20S-αΔN or T20S-K66A, as expected for a HbYX mimicking compound. This indicates that ZYA functions via the conserved HbYX-dependent mechanism, while the other compounds tested here do not appear to function by this conserved mechanism, since they do not affect the archaeal 20S. The ability of a small molecule such as ZYA to activate the proteasome nearly 50-fold with similar efficacy as the multimeric PA26 11S activator is, to our knowledge, unprecedented.

**Table 1:** Comparison of proteasome stimulation capacity and affinity of other known 20S stimulating compounds compared to ZYA.

	*V*_max_ (fold)	*K*_obs_ (μM)	*V*_max_ (95% CI)	*K*_obs_ (μM) 95% CI
AM-404	12.9	12.1	10.2 to 17.4	6.2 to 25.7
TCH-165	2.1	0.8	1.7 to 2.6	0.04 to 2.5
FLP	6.9	2.7	4.9 to 10.0	0.8 to 8.2
ZYA	48.49	970	44.32 to 55.75	963 to 977

Values derived from saturation curves in Supplementary Fig. 1B. Equilibrium coefficients were calculated from triplicates saturation curves and 95% CI are shown.

### ZYA abrogates the proteasome-inhibiting activity of three different A11+ oligomers

**Fig. 6:**
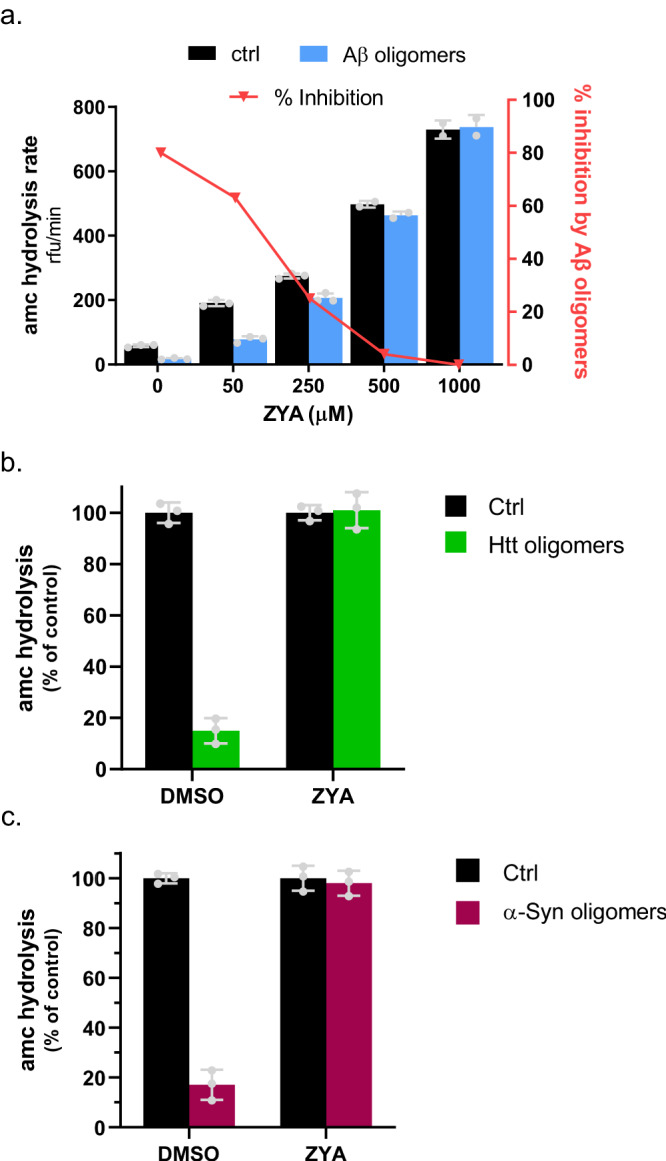
ZYA is a robust gate opening compound that can prevent inhibition by soluble oligomers. **a**. 20S proteasome (0.5 nM) activity (nLPnLD-amc hydrolysis, rfu/min) with ZYA at the indicated concentrations, with and without Aβ oligomers (0.5 μM) oligomers^21^. Rate of nLPnLD-amc hydrolysis is normalized to the control. **b**. 20S proteasomes incubated with and without α-Syn (top), and with or without ZYA (1 mM). Rate of nLPnLD-amc hydrolysis is normalized to the control. **c**. Same as b except with HttQ53 oligomers, Rate of nLPnLD-amc hydrolysis is normalized to the control. Data (means) is representative of three or more independent experiments each performed in triplicate. Error bars represent ± standard deviation.

**Fig. 7:**
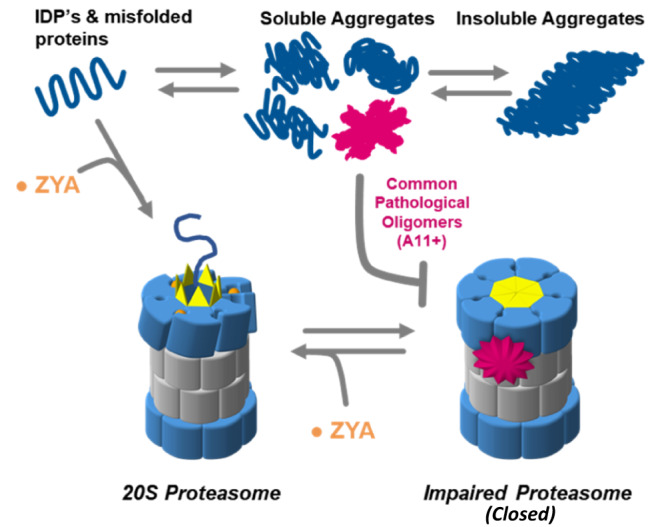
Conceptual model demonstrating rational for how small molecules like ZYA could be useful to stimulate degradation of metastable proteins associated with neurodegenerative disease, and perhaps could even circumvent inhibition by some pathological oligomers. Small molecules that function similar to ZYA, have the potential to function at two different beneficial stages of protein degradation: (1) to upregulate the degradation of unstructured proteins that could misfold, oligomerize, aggregate, and become toxic, and (2) to restore normal proteasome function to proteasomes that could be impaired by toxic oligomers.

We recently published a study demonstrating that soluble oligomers of amyloid-β (Aβ), α-synuclein, and huntingtin(Q53), which share a common tertiary conformation (A11+), allosterically inhibit 20S function by stabilizing the closed gate conformation^21^. Since ZYA stimulates gate opening, we investigated the possibility that it could also rescue the M20S from inhibition by these neurodegenerative-associated oligomers. Remarkably, when M20S activity is measured in the presence of oligomers, the addition of 1 mM ZYA completely blocks inhibition by A11 + Aβ, α-synuclein, and huntingtin(Q53) oligomers (Fig. 6a–c). Furthermore, even at 50μM, ZYA stimulates Aβ oligomer inhibited-M20S enough to bring its basal activity back to WT levels (compare control with no ZYA to Aβ oligomers at 50μM ZYA) (Fig. 6a). This is an unequivocal demonstration for the potential of small molecules to restore proteasome activity in conditions of M20S impairment. In addition, compounds that function like ZYA could also enhance the degradation of intrinsically disordered proteins (IDPs) (Fig. 7), which are often found to be the aggregation-prone proteins involved in neurodegenerative diseases^28–30^.

## Discussion

Proteinopathies are associated with many diverse human diseases (e.g., Alzheimer’s disease, cardiomyopathies, and type II diabetes) and characterized by the accumulation of intracellular (and sometimes extracellular) misfolded proteins. Many of these misfolded proteinopathy-associated proteins are IDPs^31–33^, efficiently degraded by the 20S proteasome in vitro. Why the cells’ protein degradation systems are insufficient to degrade the intracellular proteins in NDs is not clear, although many NDs have been reported to exhibit abnormally low proteasomal activity, despite relatively normal amounts of cellular proteasomes^2^. In fact, for decades now, research has implicated impairment of the proteasome system in the etiology of NDs such as Alzheimer’s and Parkinson’s^3–7^ though the specific causes remain elusive. The function of the UPS is critical for the healthy maintenance of the proteome in general, and more specifically, for neuronal synapse function (i.e., plasticity and synaptic protein turnover)^1,31^.

Based on our prior findings of oligomer impairment^21^, we generated the first multicellular organism (*C. elegans*) with a constitutively open 20S proteasome, that could not be inhibited by these toxic oligomers^32^. These worms with “hyperactive” 20S proteasomes have increased longevity and resistance to various proteotoxicities, including heat shock and oxidative damage. Moreover, increasing proteasome amount or activity in mammalian cell culture and multicellular organisms by various means has also proven feasible, and to increase the degradation of endogenous and ND related proteins^32–37^. Together, these results motivated the current study to further determine the pharmacological tractability of this mechanism to potentially treat the aforementioned pathological proteinopathies.

These findings highlight the plausibility of robustly activating the mammalian proteasome using a small molecule, designed to emulate the simplest and most fundamental aspects of the HbYX motif. Furthermore, the data clearly shows that to trigger gate opening in the 20S proteasome, complex interactions with large ring-type proteasome activators are not necessary. Instead, gate-opening can be allosterically induced by small modulators (e.g., HbYX motif), binding away from the gate within the intersubunit pockets, specifically requiring K66. Thus, this study lays the groundwork for comprehending the elemental molecular components of the HbYX motif, which are crucial for its function as a highly conserved "key" to open the 20S gate. These findings also impose size limitations on the binding pockets in the intersubunit spaces needed for allosteric gate regulation. This reduces the essential interactions to three small areas that interface with the Hb, Y side chains, and the carboxyl group of the X residue.

Our results here show that even at concentrations much less than saturating, ZYA is effective at reversing proteasome inhibition by three different ND-related proteins (Fig. 6). This inhibition reversal is obviously due to the ability of ZYA to stimulate gate-opening in a HbYX-dependent manner. We expect compounds that could similarly induce gate opening at more physiologically relevant affinities could potentially treat ND (Fig. 7). Such compounds could both stimulate the degradation of most ND-related proteins, which are typically IDPs, and simultaneously reverse proteasome impairment, which has been observed in aging. Advancements in understanding the proteasomes gate-regulatory mechanisms provide a framework for the development of small molecules that antagonize proteasome impairment, especially by oligomers, or activate its ability to degrade unstructured proteins^21^. Considering the importance of the proteasomal gate in regulating protein degradation, gate-activating compounds (such as ZYA) have promising potential as research tools to probe proteasome function in vitro and possibly in vivo to perhaps, elucidate the role of proteasome impairment in neurodegenerative disease progression.

## Methods

### Proteasome purifications

*T.acidophilum* wild type (WT) 20S, Δα_2-12,_ and all other mutant 20S proteasomes were similarly purified as described^38^, except via 8XHis tags on the C-terminus of β subunit. All 20S mutants were generated by overlapping PCR site-directed mutagenesis. The plasmid for *M.jannaschii* PAN(M74A), kindly provided by Dr. Peter Zwickl^39^, lacked a 6His-tag, and was purified as described^23^, but Tris buffers were made at 50 mM instead of 20 mM. Mammalian 20S proteasomes were isolated from bovine liver as described^40^. WT and mutant α3ΔN yeast 20S proteasomes were expressed and purified by anion-exchange chromatography as described^41^.

### Proteasome activity assays–peptide substrates

Fluorogenic substrate peptides were purchased from BostonBiochem (suc-LLVY-amc) and EZBiolabs (ac-nLPnLD-amc, ac-RLR-amc, LFP (Mca-AKVYPYPME-Dpa(Dnp)-amide)), PAN CT peptides, ZYA, and ZYA derivatives were synthesized by ABclonal. Compounds (AM-404, TCH-165, Fluspirilene (FLP)) were purchased from Cayman Chemical. Peptides and chemical compounds were dissolved in DMSO and incubated with proteasomes at indicated concentrations. The final concentration of DMSO in activity assays was 2%. Oligomers of Aβ*56, α-synuclein, and huntingtin-Q53 were prepared as described^21^. All oligomers used were recognized by the α-oligomer antibody, A11^42^. Protein concentrations were determined by Bradford assay (Thermo Scientific). To measure peptide hydrolysis, fluorogenic peptides dissolved in DMSO were used at a final concentration of 25–100 μM for Suc-LLVY-amc and Ac-nLPnLD-amc, and 3 -10 μM for LFP and Boc-LRR-amc, in 50 mM Tris (pH 7.5), 1 mM DTT. For archaeal 20S experiments the indicated concentrations of T20S and LFP peptide was added to the buffer at 45 °C, and where not indicated, 1 μg of PAN and 10 μM ATPγS (+5 mM MgCl_2_), or 2 mM ATP and 10 mM MgCl_2_, was added to the 0.1 ml of reaction buffer (sufficient to saturate the 20S particles). The amount of archaeal 20S from different purification preparations is normalized to their rate of suc-LLVY-AMC hydrolysis, whose degradation is not regulated by gating. Assays with mammalian (0.5 nM and 1 nM as indicated), yeast wild-type (2 nM), and yeast α3ΔN (0.2 nM) proteasomes were performed at 37 °C. Assays with Aβ, α-synuclein, and huntingtin-Q53 oligomers were performed as described^21^. Assays were performed for 30 min to an hour and analyzed using BioTek Gen5 Data Analysis software. The activity was measured as relative fluorescence units/minute (rfu/min), generating a curve which was used to calculate the initial velocity, according to the slope of the curves. Where indicated “Fold Activation”, fold activation was calculated by dividing the average initial velocity against the average of negative controls, with added DMSO when appropriate.

### Proteasome activity assays–protein substrates

Tau23 (gift from Eckhard Mandelkow) or β-casein (Sigma) were incubated with mammalian 20S proteasomes for the indicated time at 37^o^C. Reactions were quenched by the addition of LDS sample buffer (Invitrogen). Proteins were separated by SDS-PAGE using NuPAGE™ 4–12% Bis-Tris protein gels (Invitrogen) and visualized with Coomassie brilliant blue. Degradation of ^14^C-Casein was assessed by scintillation of acid soluble counts as described in ref. ^42^.

### Computational docking

Automated docking of the ZYA peptide mimetic in human proteasome intersubunit pocket was done with Glide from the Schrodinger Suite^43^.

### Statistics and reproducibility

Data were analyzed in Graph Pad or excel using an unpaired Student’s *t* test (Prism). For all statistical analyses, a value of *p* < 0.05 was considered significant.

### Reporting summary

Further information on research design is available in the Nature Portfolio Reporting Summary linked to this article.

## Supplementary information

Supplementary Information

Description of Additional Supplementary Files

Supplementary Data

Reporting Summary

## Data Availability

The authors declare that data supporting the findings of this study are available within the paper and its supplementary information files and are available from the corresponding author upon request. Source data for all graphs, including individual data points, can be found in an Excel file titled Supplementary Data. Full size, uncropped gels presented are found in Supplemental Fig. 3.
