# Supplementary material for: Minimal mechanistic component of HbYX-dependent proteasome activation that reverses impairment by neurodegenerative-associated oligomers
Source: Commun Biol. 2023 Jul 14;6:725. doi: 10.1038/s42003-023-05082-9 (PMC10349142; doi:10.1038/s42003-023-05082-9)
Supplement: Supplementary file 1 — Supplementary Information [file 42003_2023_5082_MOESM1_ESM.pdf]

Minimal mechanistic component of HbYX-dependent proteasome activation that reverses impairment by neurodegenerative-associated oligomers

Supplementary Information

Figures 1-3

Chuah et al.

Correspondence to David M. Smith (dmsmith@hsc.wvu.edu)

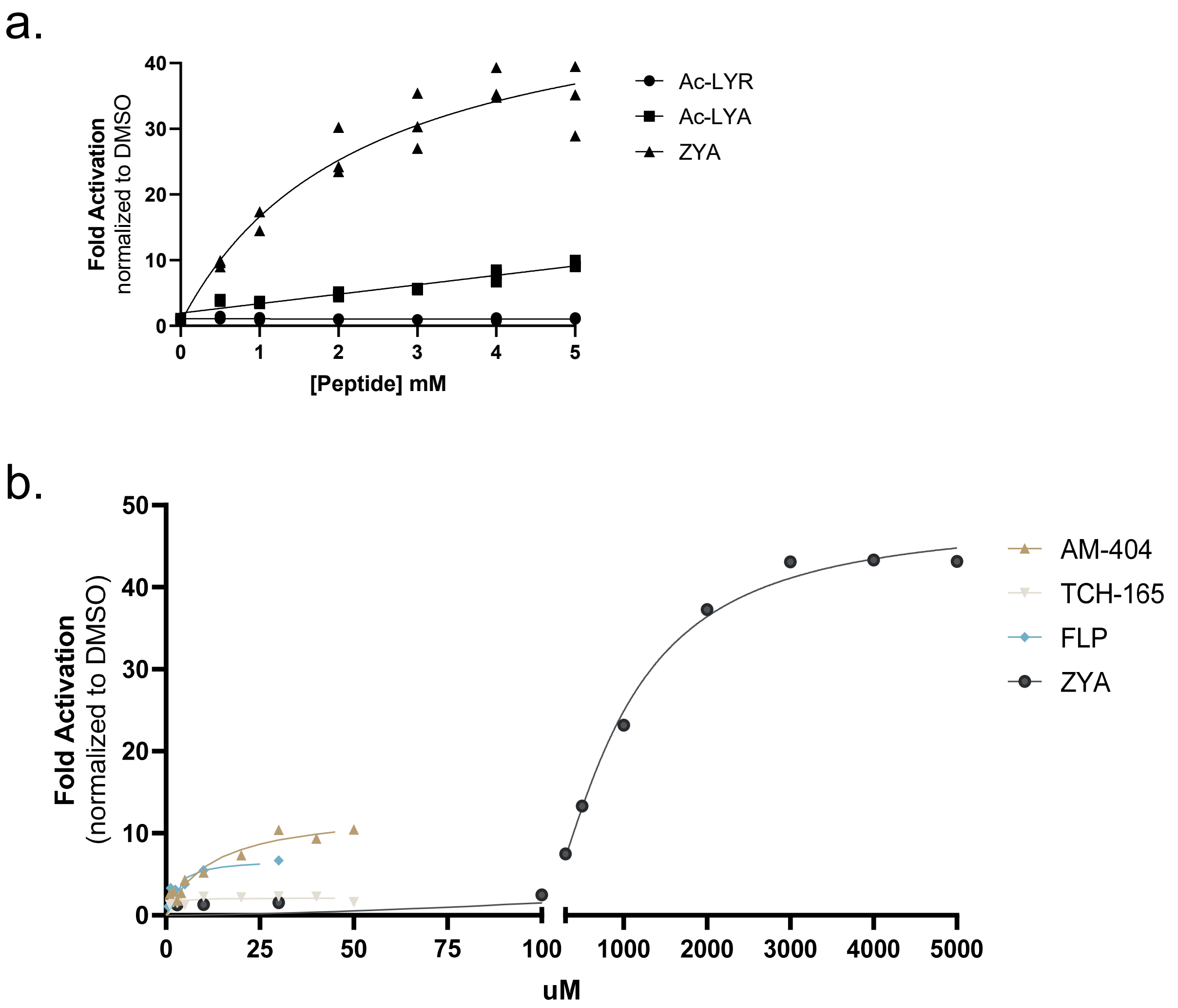

Supplemental Figure 1: ZYA's capacity to induce gate opening in mammalian 20S compared to other known small molecule activators.

a. Same as Fig 1J except peptides were incubated at indicated concentrations with mammalian 20S proteasomes (0.5nM) and nLPnLD-amc. Proteasome activity is normalized to DMSO. Experiment performed in triplicate. Error bars represent ± standard deviation.

b. Compounds incubated with mammalian 20S proteasomes (0.5nM) at indicated concentrations and suc-LLVY-amc. Proteasome activity is normalized to DMSO. Fits were generated in Prism using the Michaelis Menten equation for all compounds except ZYA, which was analyzed using the Hill equation. Data are representative of 2-3 independent experiments performed in singlicate dose response.

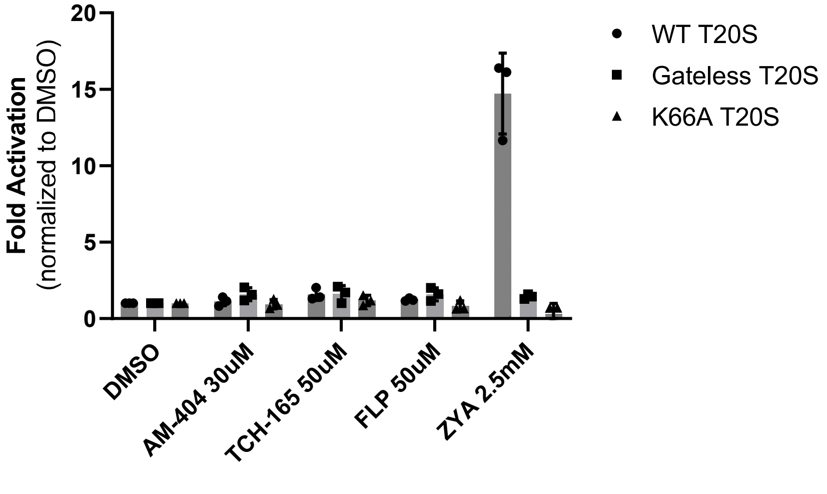

Supplemental Figure 2: ZYA's capacity to induce gate opening in wild-type and mutant archaeal 20S compared to other known small molecule activators.

Compounds incubated with 7nM T20S at saturating concentrations as indicated. Proteasome activity was measured by degradation of LFP and normalized to DMSO controls. Data was representative of 3 independent experiments performed in singlicate. Error bars represent ± standard deviation.

A

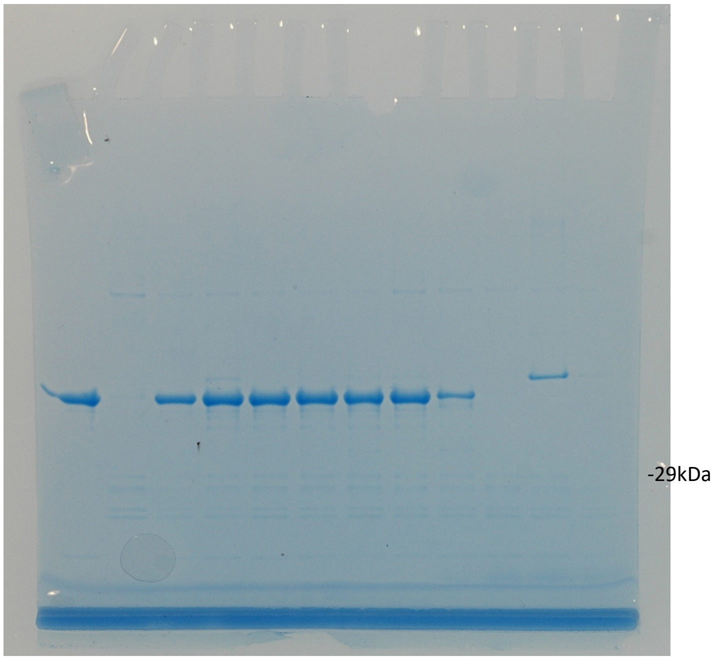

B

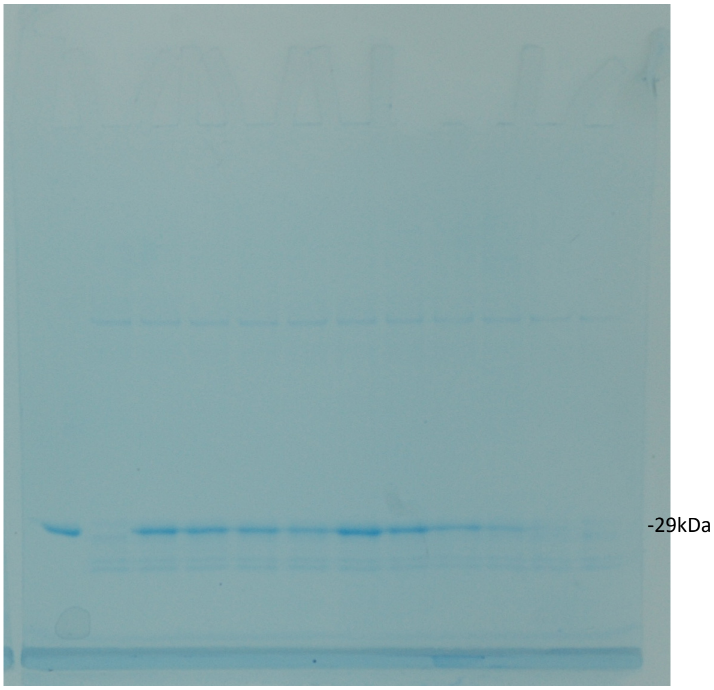

Supplemental Figure 3: Full size gels present in manuscript related to Figure 4.
a) Related to: Figure 4A
b) Related to: Figure 4B
