# Supplementary material for: Minimal mechanistic component of HbYX-dependent proteasome activation that reverses impairment by neurodegenerative-associated oligomers
Source: Commun Biol. 2023 Jul 14;6:725. doi: 10.1038/s42003-023-05082-9 (PMC10349142; doi:10.1038/s42003-023-05082-9)
Supplement: Supplementary file 2 — Description of Additional Supplementary Files [file 42003_2023_5082_MOESM2_ESM.pdf]

Description of Additional Supplementary Files

File name: Supplementary Data

Description: Source data includes excel file with tabs for each figure in manuscript. Source data for each figure includes individual data for enzymatic rates output by the Biotek Synergy plate reader.
